# Does elasticity of Achilles tendon change after suture applications? Evaluation of repair area by acoustic radiation force impulse elastography

**DOI:** 10.1186/s13018-018-0751-z

**Published:** 2018-03-02

**Authors:** Yavuz Selim Karatekin, Bedri Karaismailoglu, Gokhan Kaynak, Tahir Ogut, Atilla Suleyman Dikici, Emel Ure Esmerer, Onder Aydingoz, Huseyin Botanlioglu

**Affiliations:** 1 Department of Orthopaedics and Traumatology, Istanbul University Cerrahpasa Medical Faculty, Kocamustafapasa Cad. No:53, Fatih, Istanbul, Turkey; 2 Department of Radiology, Istanbul University Cerrahpasa Medical Faculty, Kocamustafapasa Cad. No: 53, Fatih, Istanbul, Turkey

**Keywords:** Achilles tendon rupture, Surgical repair, Modified Kessler method, Krackow method, Acoustic radiation force impulse, Elastography, Elasticity, Shear wave velocity

## Abstract

**Background:**

Achilles tendon injuries are one of the most common tendon injuries. Surgical treatment is preferred in young and active patients. Although there are studies which evaluate the repair area with magnetic resonance imaging and ultrasonography after surgical treatment, there are very few studies which analyzes the elasticity of the tendon by quantitative methods. ARFI (acoustic radiation force impulse) elastography is a simple and non-invasive method that can quantitatively measure the elasticity of the soft tissues. Our study aims to evaluate the elasticity in the repair area of the surgically treated Achilles tendons, compare them to the non-injured side, and evaluate the effect of the suture method to the elasticity of the repaired tendons by using ARFI elastography.

**Methods:**

In our retrospectively designed study, 19 patients who underwent surgical treatment with Krackow and modified Kessler suture methods after the Achilles tendon rupture between 2006 and 2014 were included. Shear wave velocity (SWV) of the repaired and non-injured Achilles tendons were measured by ARFI elastography in four different positions of the ankle.

**Results:**

It was determined that SWV in the surgically repaired tendons were significantly higher in each four different position of the ankle, compared to the non-injured side (*p* < 0.01), indicating less elasticity in the repaired tendons. There was no statistically significant difference between the SWV of Krackow and modified Kessler suture method groups at four different positions of the ankle (*p* > 0.05). AOFAS Ankle-Hindfoot, VISA-A, VAS, and FAOS scores were not also statistically different between these two suture methods (*p* > 0.05).

**Conclusions:**

In the repaired Achilles tendon, there is a decrease in the elasticity compared to the non-injured side. The functional and elastographic results of Krackow and modified Kessler suture methods are similar in long-term follow-ups of the patients.

## Background

The Achilles tendon is the strongest and largest tendon in the human body and also among the most commonly injured tendons [1]. The parallel bundle of type I collagen fibers forms the main structure of the Achilles tendon. This feature increases the tendon’s elasticity and allows the tendon to lengthen and shorten during daily activities. During the healing process, type III collagen content increases and the tendon becomes heterogeneous [2]. In addition, it is known that the tendon gets stiffer and loses its elasticity as a result of the fibrosis in the tendon and the surrounding area after healing [3]. For this reason, it is important to evaluate the repaired Achilles tendons to reveal the changes in the elasticity and structural properties.

Treatment of Achilles tendon rupture (ATR) can be done both conservatively and surgically, but the debate about the best choice of the treatment still continues. Several suture methods have been described for the repair of ATR. The most commonly used methods are Krackow and modified Kessler suture methods, and the studies have shown that no significant functional difference exists between these methods [4–6]. Comparisons between these two methods are mostly about the suture strength and the tendon structure after repair, but there is no study comparing the stiffness and elasticity of the repaired tendons with these two methods [4–6].

Acoustic radiation force impulse (ARFI) elastography, which is one of the new imaging methods in recent years, is based on the principle of measuring the velocity of the sound waves advancing on the tissues. In soft tissues, shear wave velocity (SWV) is low and displacement amplitude is high, while in hard tissues, SWV is high and displacement amplitude is low [7]. Therefore, it is a quantitative measure of the elasticity of the soft tissues such as muscles and tendons [8]. In addition, the ability to measure the elasticity of the tissues quantitatively without compressing the tissue minimizes the intra- and inter-observer variability between radiologists and makes the results more objective and reliable.

There are numerous biomechanical and clinical investigations after Achilles tendon repair, but there are quite few sources evaluating the elasticity of the repaired tendon with the help of imaging methods. The purpose of this study is to evaluate the elasticity of the surgically treated tendons after ATR and to compare the elasticity of the Achilles tendons repaired with different suture methods.

## Methods

After obtaining approval from the local Ethics Committee, the patients over 18 years of age who were operated due to traumatic ATR between 2006 and 2014 were retrospectively reviewed. To minimize the effect of healing process which we know that might take over the course of 1 year, the patients with less than 4-year follow-up were not included in the study [9]. Patients who had previous oral, IM or IV steroid use, rheumatologic diseases, malignant tumors, diabetes mellitus, hypercholesterolemia, spondyloarthropathies, fluoroquinolone use, post-operative re-rupture, ankle arthrosis, ankle impingement syndrome, or deep surgical site infection were not included in the study. Two female patients were excluded from the study due to their pregnancy and its possible effect to the tendon elasticity [10]. Nineteen patients met the inclusion criteria for the study and returned for clinical evaluations. The power analysis revealed that the sample size for each group should be at least seven patients, which is less than the number of patients in our groups, suggesting that the sample size is adequate. All of the patients were males. Eighteen patients were operated unilaterally while one patient was operated bilaterally. The patient who underwent bilateral repair was included in the comparisons according to suture method but was not included in the comparisons between the repaired and the non-injured side. The functional evaluations of the patients were made by visual analogue scale (VAS), American Orthopedic Foot and Ankle Score (AOFAS) Ankle-Hindfoot, FAOS Foot and Ankle Outcome Score (FAOS), and VISA-A scoring (Victorian Institute of Sports Assessment – Achilles).

The measurements were made by the same musculoskeletal radiologist. Evaluations were performed using a Siemens Acuson S2000 ultrasound system (Siemens Medical Solutions, Mountain view, CA, USA), and a 4–9 MHz transducer (9L4) was equipped for B-mode ultrasound and ARFI elastography examinations.

**Fig. 1:**
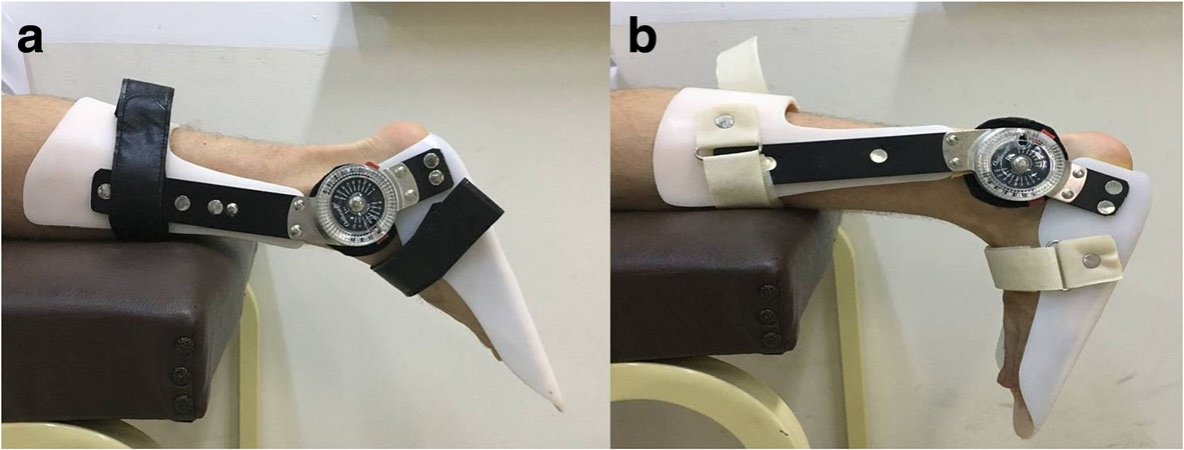
Angle adjustable short leg brace with a hinge centered over the ankle to standardize the measurements. 30° plantar flexion (**a**) and 30° dorsiflexion (**b**). *The patient provided written informed consent for print and electronic publication of the figures*

Patients were placed in the supine position and their knee at full extension while the ankles of the patients were evaluated in four different positions: plantar flexion, neutral position, and 15° and 30° dorsiflexion. To standardize the positions, a hinged angle adjustable short leg brace was used in which the Achilles region was left open (Fig. 1). Measurements were made in the musculoskeletal mode of the ultrasound device. The probe was placed parallel to the muscle fibers on the longitudinal axis, and measurements were made between 3 cm above and below the Achilles tendon repair area where the clearest and homogeneous image was obtained. The tendon diameters were measured to determine the middle part of the tendon and measurements were made at this level. No measurement was made from the areas that were artifacted by unabsorbed sutures.

**Fig. 2:**
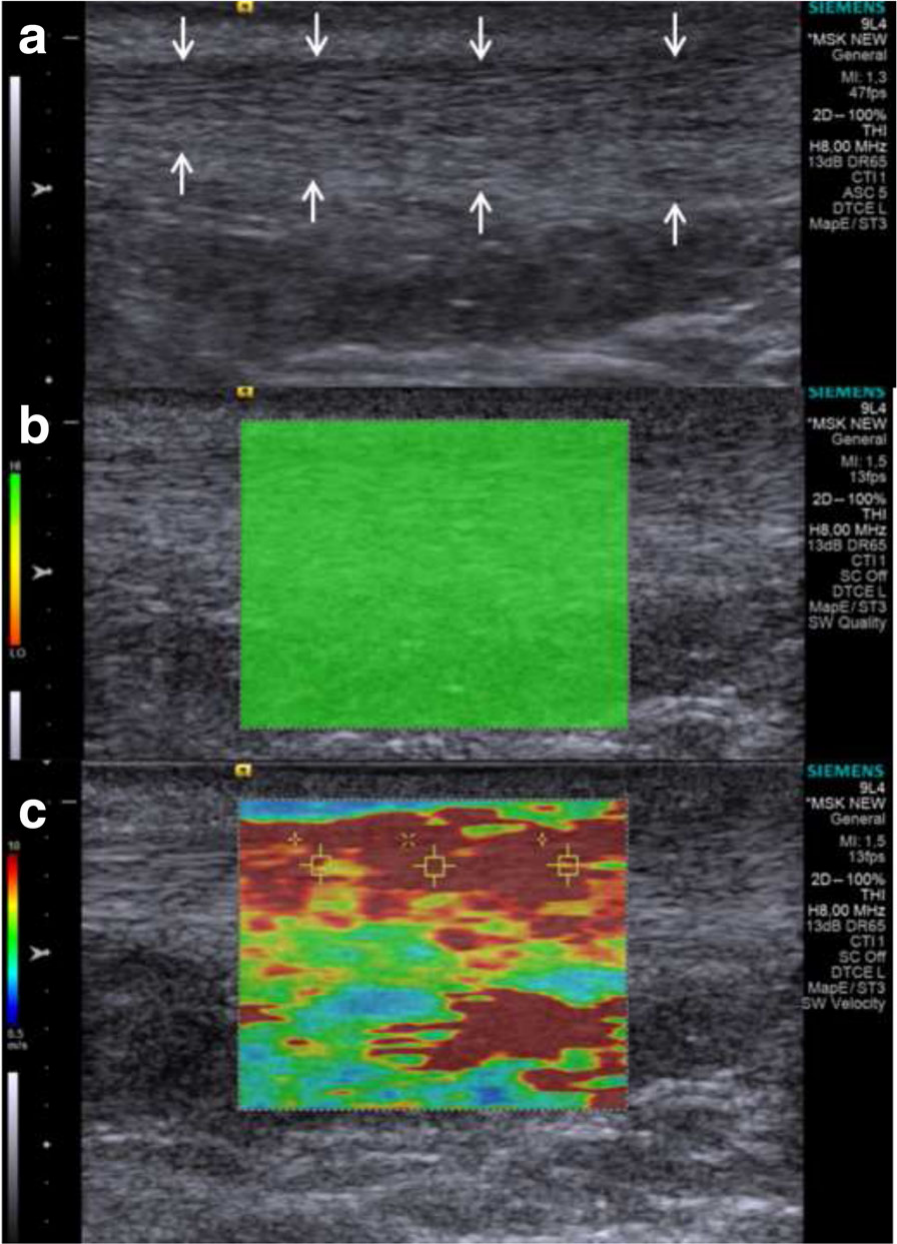
B-mode ARFI image (**a**), ARFI elastography mapping image without any artifact indicating a homogenous area (**b**), and elastographic measurement (**c**)

First of all, the rectangular area was mapped by coloring according to the stiffness of the tissue in B-Mode of ARFI device. The blue color on the scale shows the softest region, while the red shows the hardest region. Radiologist recorded sections from three regions (region of interest) where it is considered to be the most homogeneous within this rectangle and the SWVs given by the program were recorded (Fig. 2). SWV value was expressed in meters per second. The range for SWV was 0–9 m/s. For each tendon, three measurements were performed. Analyses were made by taking the average of the values in the examined sections.

Statistical analyses were performed using SPSS program, version 22.0 (SPSS Inc.). Mann-Whitney *U* test was used to test whether the measurements in the Krackow and modified Kessler suture method groups were different. Wilcoxon signed-rank test was used for the purpose of examining differences between the measurements of the repaired and the non-injured tendons. Standard deviation (SD) was used as a measure of variability. The results were presented with two decimal places. *P* values less than 0.05 were considered statistically significant.

## Results

Ten unilaterally operated patients underwent surgical repair with Krackow suture method, while one bilateral and eight unilaterally operated patients underwent modified Kessler suture method. The mean age of the patients included in the study was 42.10 years (range 28–56, SD ± 8.96). The mean age of the patients who underwent Krackow suture method was 37.2 years (range 28–45, SD ± 7.57), while the mean age of the patients who underwent modified Kessler suture method was 47.1 years (range 40–56, SD ± 7.67). The mean follow-up was 84.2 months (range 48–156, SD ± 14.37). The mean follow-up of the patients who underwent Krackow suture method was 82.4 months (range 48–148, SD ± 15.43), while the mean age of the patients who underwent modified Kessler suture method was 86.1 months (range 54–156, SD ± 17.22).

Achilles tendons of the patients operated with Krackow and modified Kessler methods were compared with ARFI elastography in four different positions of the ankle, and no significant difference was found between the groups (*p* > 0.05) (Table 1).

**Table 1:** Comparison of average SWVs of surgically repaired Achilles tendons according to the suture method by Mann-Whitney *U* test and associated *p* values

Tendon	Suture method	*N*	SWV ± SD (m/s)	*p* value
Repaired P	Krackow	10	6.48 ± 0.78	0.55
	Modified Kessler	10	6.26 ± 0.62	
Repaired N	Krackow	10	7.35 ± 0.70	0.42
	Modified Kessler	10	7.28 ± 0.64	
Repaired D 15°	Krackow	10	8.23 ± 0.58	0.39
	Modified Kessler	10	8.42 ± 0.37	
Repaired D 30°	Krackow	10	9.24 ± 0.46	0.43
	Modified Kessler	10	9.32 ± 0.20	

There was no statistically significant differences between the groups

*SWV* shear wave velocity, *P* plantar flexion, *N* neutral, *D* dorsiflexion

There was no significant difference (*p* > 0.05) between the functional results (VAS, AOFAS Ankle- Hindfoot, VISA-A) of the Krackow and modified Kessler methods (Table 2).

**Table 2:** Comparison of average functional scores of the patients according to the suture method by Mann-Whitney *U* test and associated *p* values

Questionnaire	Suture method	*N*	Score	SD	*p* value
VAS	Krackow	10	0.30	0.67	0.99
	Modified Kessler	10	0.41	0.61	
AOFAS Ankle-Hindfoot	Krackow	10	97.13	4.32	0.87
	Modified Kessler	10	96.87	4.13	
VISA-A	Krackow	10	98.51	2.32	1.12
	Modified Kessler	10	98.12	2.51	

There was no statistically significant differences between the groups

*VAS* Visual Analogue Scale, *AOFAS* American Orthopedic Foot and Ankle Score, *VISA-A* Victorian Institute of Sports Assessment – Achilles

The scores of five subscales of FAOS were similar between the two suture method groups (*p* > 0.05) (Table 3).

**Table 3:** Comparison of average FAOS (Foot and Ankle Outcome Score) of the patients according to the suture method by Mann-Whitney *U* test and associated *p* values

	Suture method	*N*	Score (%)	SD	*p* value
Pain (%)	Krackow	10	97.78	3.66	0.27
	Modified Kessler	10	99.17	1.34	
Symptoms (%)	Krackow	10	97.50	2.41	0.57
	Modified Kessler	10	98.22	3.03	
Daily activities (%)	Krackow	10	99.85	0.46	0.49
	Modified Kessler	10	99.56	0.71	
Sports and recreational activities (%)	Krackow	10	95.50	8.32	0.15
	Modified Kessler	10	99.50	1.58	
Quality of life (%)	Krackow	10	86.88	11.20	0.90
	Modified Kessler	10	87.50	11.41	

There were no statistically significant differences between the groups

**Fig. 3:**
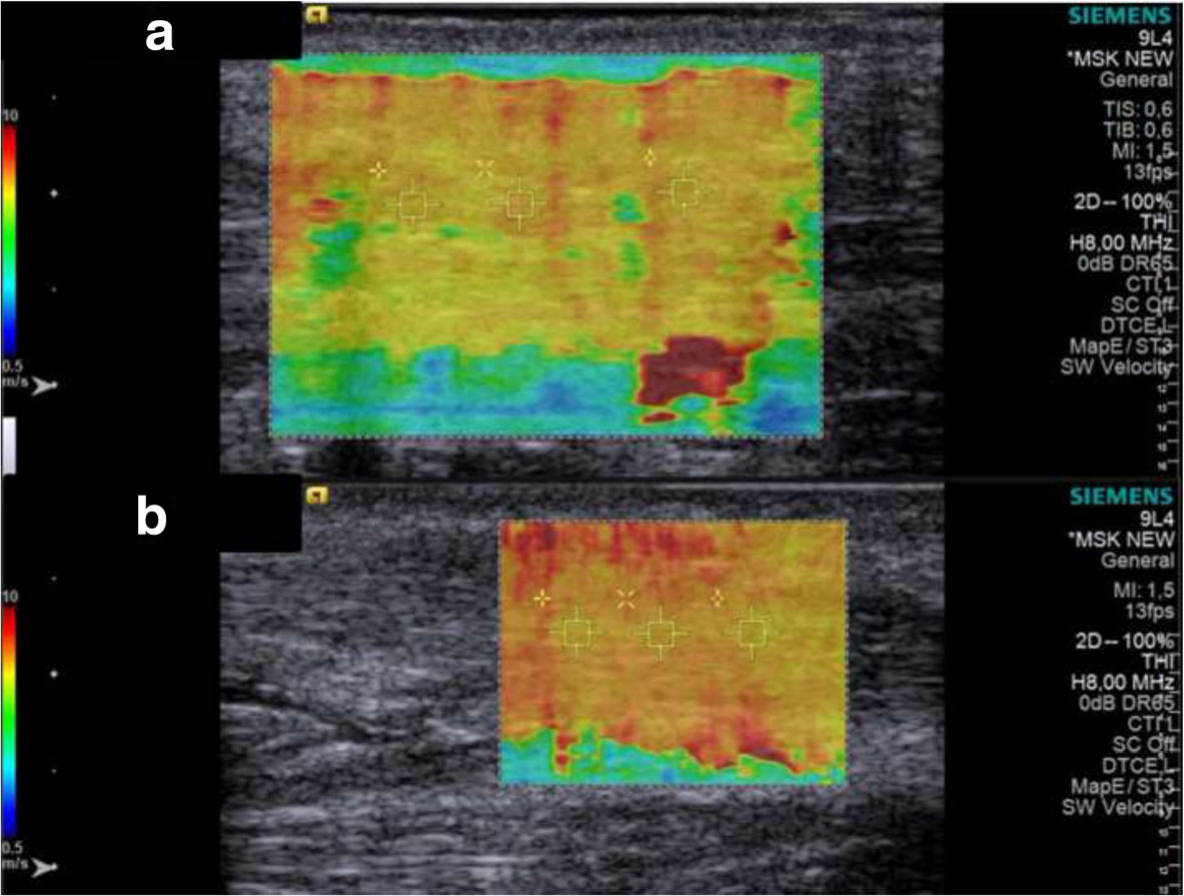
Elastographic measurements from a surgically repaired Achilles tendon (**a**, **b**)

The repaired and non-injured Achilles tendons of the patients were compared with ARFI elastography, and the measurements on the repair side were significantly higher in all four different positions of the ankle (*p* < 0.05) (Table 4) (Fig. 3).

**Table 4:** Comparison of the ARFI values of healthy and repaired tendons by Wilcoxon signed-rank test

Measurement	N	SWV ± SD (m/s)	*p* value
Repaired P	18	6.37 ± 0.70	0.01*
Healthy P	18	3.93 ± 0.81	
Repaired N	18	7.31 ± 0.65	0.01*
Healthy N	18	5.64 ± 0.89	
Repaired D 15°	18	8.32 ± 0.49	0.01*
Healthy D 15°	18	7.35 ± 0.70	
Repaired D 30°	18	9.28 ± 0.35	0.01*
Healthy D 30°	18	8.48 ± 0.54	

Repaired tendons had significantly higher values compared to the healthy tendons, indicating less elasticity in the repaired tendons

*SWV* shear wave velocity, *P* plantar flexion, *N* neutral, *D* dorsiflexion

*p < 0.05

## Discussion

In this study, we found out that the repaired Achilles tendon has lower elasticity compared to the non-injured tendon, but the Krackow and modified Kessler suture methods does not have different effects on the elasticity.

In the treatment of ATR, numerous treatment approaches have been identified but there is no consensus about the gold standard treatment method. The healing process always includes fibrosis in the tendon and the surrounding area, independent from the treatment method. As a result, the increased fibrotic tissue in the healing zone increases the stiffness of the tendon [3]. This fact also supports our results which suggests that the elasticity of the repaired Achilles tendons is significantly lower than the non-injured Achilles tendons.

Although the non-operative management with functional bracing remains as one of the valuable treatment options due to the studies showing similar re-rupture rates with avoidance from possible surgical complications, the surgical repair is widely preferred especially in young patients for faster recovery and early return to the physical activities [11]. There are various techniques for the surgical repair of ATR including open, minimal invasive, and percutaneous techniques. In the literature, numerous studies exist comparing the outcomes of these techniques but it is not possible to determine a gold standard. In an in vitro study by Hockenbury and Johns on fresh-frozen cadavers, it was shown that the open repair provides two-times more strength and less risk of sural nerve entrapment compared to the percutaneous technique [12]. However, Hsu et al. did not find any significant difference between these two techniques in terms of postoperative complications in their cohort of 270 patients, while Cretnik et al. obtained less complications in the patients treated with percutaneous technique compared to the open repair group in their cohort of 237 patients [13, 14].

Bhattacharyya and Gerber claimed that the open repair has increased rate of wound complications and need for the postoperative analgesia compared to the minimal invasive technique [15]. In another comperative study by Aktas and Kocaoglu, open and minimal invasive techniques yielded similar functional outcomes while the open repair group had a higher complication rate [16]. In their meta-analysis, Del Buono et al. concluded that minimally invasive and open surgery of the Achilles tendon are grossly equivalent in terms of functional outcomes, but the recent evidence is that minimally invasive surgery results in a lower rate of complications than open surgery [17].

Several methods have been described for the repair of the Achilles tendon. Kessler, Krackow, Bosworth, Bunnell, modified Kessler, Lynn’s, and Lindholm methods are some of these described techniques in the literature [17]. Krackow and modified Kessler methods, which are the methods that had been used in this study, are two of the most commonly used end-to-end repair methods. Modified Kessler method consists of grasping loops at both medial and lateral side of the tendon ends, and the knot is tied in the repair site. Krackow method includes a series of locking loops that reinforce one another going through both medial and lateral side of the tendon ends. The knot of the suture is also tied in the repair site. Although there is no significant functional difference in the various comparative studies comparing these suture methods [4–6], there are also some publications that indicate that the Krackow suture method is stronger than the modified Kessler suture method biomechanically [18, 19]. However, when we look at our findings, there was no difference between the two techniques in terms of the change in the elasticity and also the functional outcomes.

In the literature, there are many studies evaluating the Achilles tendon’s structural properties after repair with the aid of both histological, biomechanical, and imaging studies, but there are quite few researches aimed at evaluating elastic properties of the tendon. The elastographic evaluation of the soft tissues is a trending topic in musculoskeletal radiology, and the Achilles tendon is one of the main interests of the researchers. Some studies have shown that elastic properties of the Achilles tendon correlates with the clinical outcomes and can be a useful tool to follow the effect of the treatment [20, 21]. Zhang et al. investigated the correlation between elasticity of the Achilles tendon and functional outcomes at 12, 24, and 48 months postoperatively in 26 patients who underwent surgical repair after ATR. They concluded that SWE can provide biomechanical information for evaluating the mechanical properties of healing Achilles tendon and predict Achilles tendon function [20]. Dirrichs et al. also found that SWE findings and clinical scores correlates strongly in patients with symptomatic tendinopathies (17 Achilles, 15 patellar, and 15 humeral-epicondylar tendons) [21].

In some elastographic studies of the Achilles tendon, it has also been showed that the elasticity of the tendon decreases by age [7, 22]. In a series of 56 healthy individuals evaluated by ARFI elastography, Ruan et al. found out that the elasticity of healthy Achilles tendon decreases with increasing age, even in subjects without disease and long-term heavy load lifting [7]. In a sonoelastographic study by Turan et al., the authors revealed an increased tendon stiffness in elderly subjects and concluded that this might be a predisposing factor for the high prevalence of Achilles tendinopathies observed in elderly subjects [22]. In our study, the mean age of the patients in the modified Kessler suture method group was significantly higher (~ 10 years) than the mean age of the patients in the Krackow suture method group. But this difference was also significantly low compared to the aforementioned studies which have groups with age differences more than 25 years. Therefore, the age difference in our groups might not have affected the results significantly.

The functional results of the patients were very good in all questionnaires including AOFAS Ankle-Hindfoot, VAS, VISA-A, and all subscales of FAOS. However, “The Quality of Life” subscale of FAOS was relatively low compared to the other subscales. The anamnesis of the patients revealed that there is a sense of insecurity towards the repaired Achilles tendons, even though the patients have no clinical complaints. We think that this is the main reason behind the relatively low “The Quality of Life” score in FAOS. The less elasticity of the repaired tendon may have led to an insecurity through a natural defense mechanism to prevent re-injuries.

Our study has several limitations. The number of patients in our study was limited, and the age distribution of patient groups was not homogenous. This situation might have affected our results since the elasticity of the Achilles tendon decreases by age, according to some studies [7, 22]. Retrospective design of the study and non-standardized rehabilitation protocol also negatively affects the strength of our study. Having only male patients in the study group also restricts the generalization of our results to the entire population. However, in some studies including the Shear Wave Elastography assessments, the elasticity of the Achilles tendon was not different between men and women groups [7, 23]. Finally, our study did not include a patient group who were conservatively treated. Conducting studies which compares the elasticity of the conservatively and the surgically treated Achilles tendons may bring new information to the management of ATRs.

Despite these limitations, this study has several strengths. All clinical evaluations were performed by the same physician, and all radiologic evaluations were performed by the same radiologist which reduced the possible inter-observer variability. To our knowledge, our study is the first to evaluate the elasticity of the repaired and the non-injured Achilles tendons and compare the results according to the suture method.

## Conclusion

In our study, the repaired Achilles tendon ruptures were retrospectively evaluated by ARFI elastography and the elasticity on the repair site was found to be less compared to the non-injured side in four different positions of the ankle. At the same time, there was no significant difference between Krackow and modified Kessler methods in ARFI elastography and clinical evaluations. These results suggest that the repair method should be left to surgeon preference. The SWV values of the repaired and the non-injured tendons obtained in this study can be used as data for the future studies with ARFI elastography. More comprehensive studies investigating the help of elastography on evaluating the tissue-healing and deciding the timing of immobilization, weight-bearing, and rehabilitation can help the surgeons to have more patient-based treatment and rehabilitation approaches.
